# Highly optical transparency and thermally stable polyimides containing pyridine and phenyl pendant

**DOI:** 10.1080/15685551.2017.1351766

**Published:** 2017-07-11

**Authors:** Jianan Yao, Chunbo Wang, Chengshuo Tian, Xiaogang Zhao, Hongwei Zhou, Daming Wang, Chunhai Chen

**Affiliations:** ^a^ National & Local Joint Engineering Laboratory for Synthesis Technology of High Performance Polymer, Key Laboratory of High Performance Plastics, Ministry of Education, College of Chemistry, Jilin University, Changchun, P. R. China

**Keywords:** Polyimides, pyridine, kinky structure, optical transparency, thermal stability

## Abstract

In order to obtain highly optical transparency polyimides, two novel aromatic diamine monomers containing pyridine and kinky structures, 1,1-bis[4-(5-amino-2-pyridinoxy)phenyl]diphenylmethane (BAPDBP) and 1,1-bis[4-(5-amino-2-pyridinoxy)phenyl]-1-phenylethane (BAPDAP), were designed and synthesized. Polyimides based on BAPDBP, BAPDAP, 2,2-bis[4-(5-amino-2-pyridinoxy)phenyl]propane (BAPDP) with various commercial dianhydrides were prepared for comparison and structure-property relationships study. The structures of the polyimides were characterized by Fourier transform infrared (FT-IR) spectrometer, wide-angle X-ray diffractograms (XRD) and elemental analysis. Film properties including solubility, optical transparency, water uptake, thermal and mechanical properties were also evaluated. The introduction of pyridine and kinky structure into the backbones that polyimides presented good optical properties with 91–97% transparent at 500 nm and a low cut-off wavelength at 353–398 nm. Moreover, phenyl pendant groups of the polyimides showed high glass transition temperatures (*T*_*g*_) in the range of 257–281 °C. These results suggest that the incorporating pyridine, kinky and bulky substituents to polymer backbone can improve the optical transparency effectively without sacrificing the thermal properties.

## 1. Introduction

Polyimides are well known for their excellent thermal stability, mechanical properties, chemical resistance, and electrical properties and have been used in the fields of adhesives, composites, fibres, films, and electronics [1–7]. Fully aromatic polyimides have rigid chains and strong interactions derived from intra and interchain charge transfer complex (CTC), which lead to their poor solubility and low transmittance [8–10]. Thus, new polyimides with aliphatic, asymmetrical and flexible linkages, bulky and kinky substituents incorporated into the backbone have been developed to improve solubility, processability and optical transparency [11–18]. However, the introduction of these groups often leads to the loss of thermal stability to some extent.

To overcome these problems, we designed and synthesized a series of novel polyimides based on pyridine. Pyridine are a class of n-type heterocyclic compounds with high thermal stabilities, and because of this, they have been a key molecule in constructing functional materials [19]. Moreover, pyridine groups possess relatively high mole refraction as compared to phenyl unit which leads to the polyimides containing pyridine showed high optical transparency [20]. The polarizability derived from the nitrogen of the pyridine ring can improve the polyimides solubility in organic solvents too [21]. It has been reported that polyimides synthesized with commercial dianhydrides and diamines containing pyridine units have improved solubility [22–24]. In the previous work, we have studied on the structure-property relationships of pyridine-polyimides containing –(CF_3_)_2_, –O–, –SO_2_–, –S–, –CO–, cyclohexane and biphenyl groups. These polyimides showed highly optical transparency, low dielectric constants, good thermal stability, excellent mechanical properties, respectively [25–29].

In this work, we have synthesized the diamines with pyridine and kinky structures derived from phenyl pendants. The introduction of pyridine can improve the optical transparency, and the phenlic pendant as kinky structure disrupt the formation of CTC without the sacrificing of thermal stability [9]. Herein, two novel diamine monomers, 1,1′-bis[4-(5-amino-2-pyridinoxy)phenyl]diphenylmethane (BAPDBP) and 1,1′-bis[4-(5-amino-2-pyridinoxy)phenyl]-1-phenylethane (BAPDAP) were synthesized and characterized. In addition, 2,2′-bis[4-(5-amino-2-pyridinoxy)phenyl]propane (BAPDP) was prepared for comparison [25]. We prepared the polyimides using different diamines (BAPDBP, BAPDAP, BAPDP and 6FDA) as substituents in the backbone to investigate their effect on thermal stability, optical transparency, solubility, water uptake and mechanical properties. A series of polyimides were also prepared from BAPDBP and three commercially available dianhydrides, and their properties were investigated for promising potential application.

## 2. Materials and methods

### 2.1. Materials

3,3′,4,4′-Oxydiphthalic anhydride (ODPA) was obtained from Beijing Jiaohua Company. 3,3′,4,4′-Biphenyl tetracarboxylic dianhydride (s-BPDA) were supplied by Chriskev Company Inc. 4,4′-(Hexafluoroisopropylidene)-diphthalic anhydride (6FDA) was supplied by Sinopharm Chemical Reagent Beijing Co. Ltd. The aromatic dianhydrides were all dried in a vacuum oven at 200 °C for 10 h prior to use. 2,2-Bis[4-(4-aminophenoxy)phenyl]propane was supplied by Sunlight Pharmaceutical Company (BAPP). 2-Chloro-5-nitropyridine, 4,4′-(propane-2,2-diyl)diphenol (Acros), 4,4′-(1-phenylethane-1,1-diyl)diphenol, 4,4′-(diphenylmethylene)diphenol, potassium carbonate (K_2_CO_3_), 10% palladium on charcoal (Pd/C), and 80% hydrazine monohydrate were obtained from Acros and used as received. N,N-dimethylformamide (DMF) and N,N-dimethylacetamide (DMAc) were dried with magnesium sulfate, purified by vacuum distillation and stored over 4 Å molecular sieves prior to use. All other chemicals were used without further purification.

### 2.2. Monomer synthesis

**Scheme 1.**
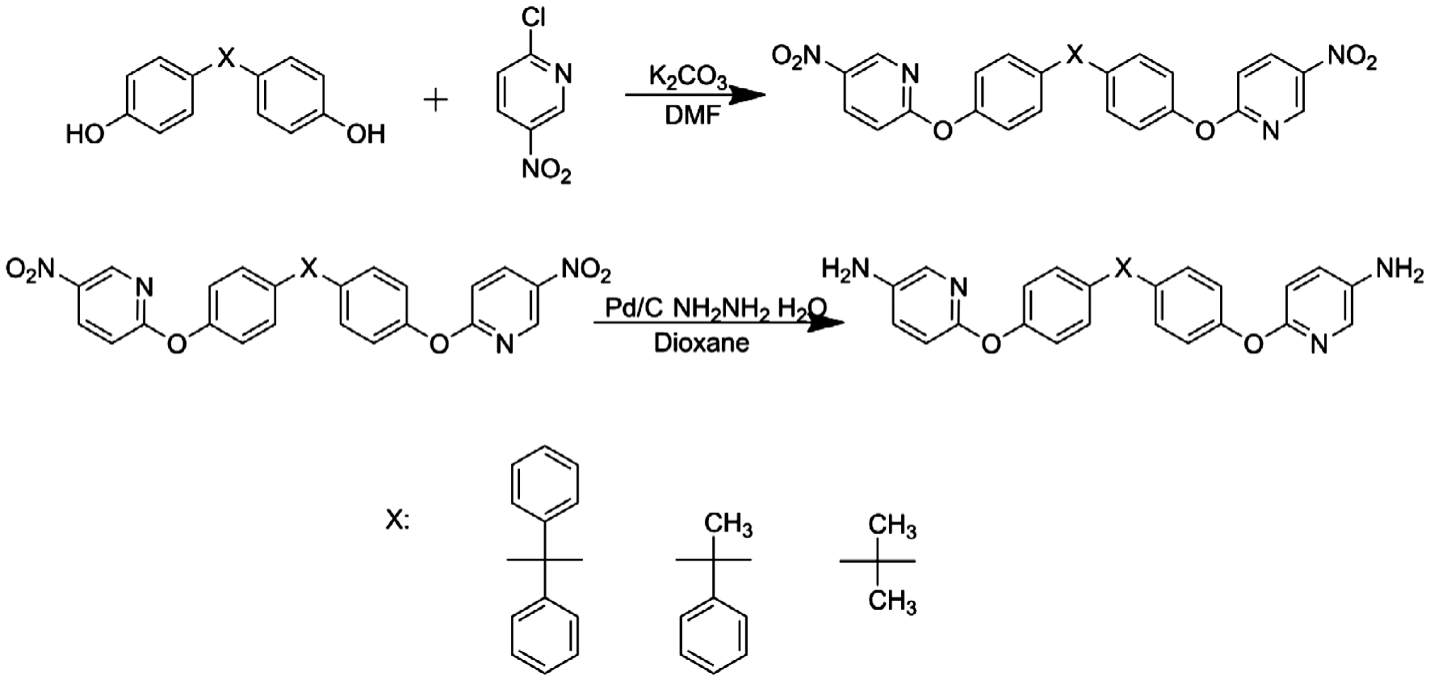
Synthesis of diamine monomers.

The procedure to synthesize BAPDBP, BAPDAP, BAPDP were performed according to the literature [25]. The route of diamine monomers were shown in Scheme 1.

#### 2.2.1. 1,1′-bis[4-(5-nitro-2-pyridinoxy)phenyl]diphenylmethane (BNPDBP)

A 250 mL flask containing 4,4′-(diphenylmethylene)diphenol (6.00 g, 17 mmol), 2-chloro-5-nitropyridine (6.47 g, 40.80 mmol), 80 mL DMF, and potassium carbonate (5.64 g, 40.80 mmol) was fitted with a mechanical stirrer, condenser, nitrogen inlet and thermometer. After 30 min of stirring at room temperature, the mixture was continuously reacted at 75 °C for 6 h. Then, the reaction mixture was cooled and poured into 500 ml of distilled water. The precipitated product was filtered off and washed with water until it was neutral. The crude product was recrystallized from DMF/water and then dried under vacuum at 80 °C for 10 h to yield 8.8 g (71%).

#### 2.2.2. 1,1′-bis[4-(5-nitro-2-pyridinoxy)phenyl]-1-phenylethane (BNPDAP)

1,1′-bis[4-(5-nitro-2-pyridinoxy)phenyl]-1-phenylethane was prepared in the same way. Yield: 65%.

#### 2.2.3. 2,2′-bis[4-(5-nitro-2-pyridinoxy)phenyl]propane (BNPDP)

The synthesis of 2,2′-bis[4-(5-nitro-2-pyridinoxy)phenyl]propane was conducted in the same way. Yield: 90%.

#### 2.2.4. 1,1′-bis[4-(5-amino-2-pyridinoxy)phenyl]diphenylmethane (BAPDBP)

Under nitrogen protection, a mixture of 7.5 g (12.57 mmol) of BNPDBP, 3 g of Pd/C catalyst, and 150 mL of dioxane was placed into a 250 mL three-necked flask equipped with a dropping funnel, and reflux condenser. The mixture was stirred under reflux for 30 min, and then 25 mL of hydrazine hydrate was added dropwise over 2 h, followed by 6 h of reflux. The resulting mixture was filtered while hot to remove the catalyst and the filtrate was subsequently concentrated and poured into 500 mL of deionized water to produce a precipitate, which was washed with water. After recrystallization from dioxane/water, the product was dried under vacuum at 80 °C for 10 h to yield 5.8 g (77%).

#### 2.2.5. 1,1′-bis[4-(5-amino-2-pyridinoxy)phenyl]-1-phenylethane (BAPDAP)

The synthesis of 1,1-bis[4-(5-amino-2-pyridinoxy)phenyl]diphenylmethane was conducted in the same way. Yield: 80%.

#### 2.2.6. 2,2′-bis[4-(5-amino-2-pyridinoxy)phenyl]propane (BAPDP)

The synthesis of 2,2-bis[4-(5-amino-2-pyridinoxy)phenyl]propane was conducted in the same way. Yield: 85%.

### 2.3. Preparation of polyimide films

**Scheme 2.**
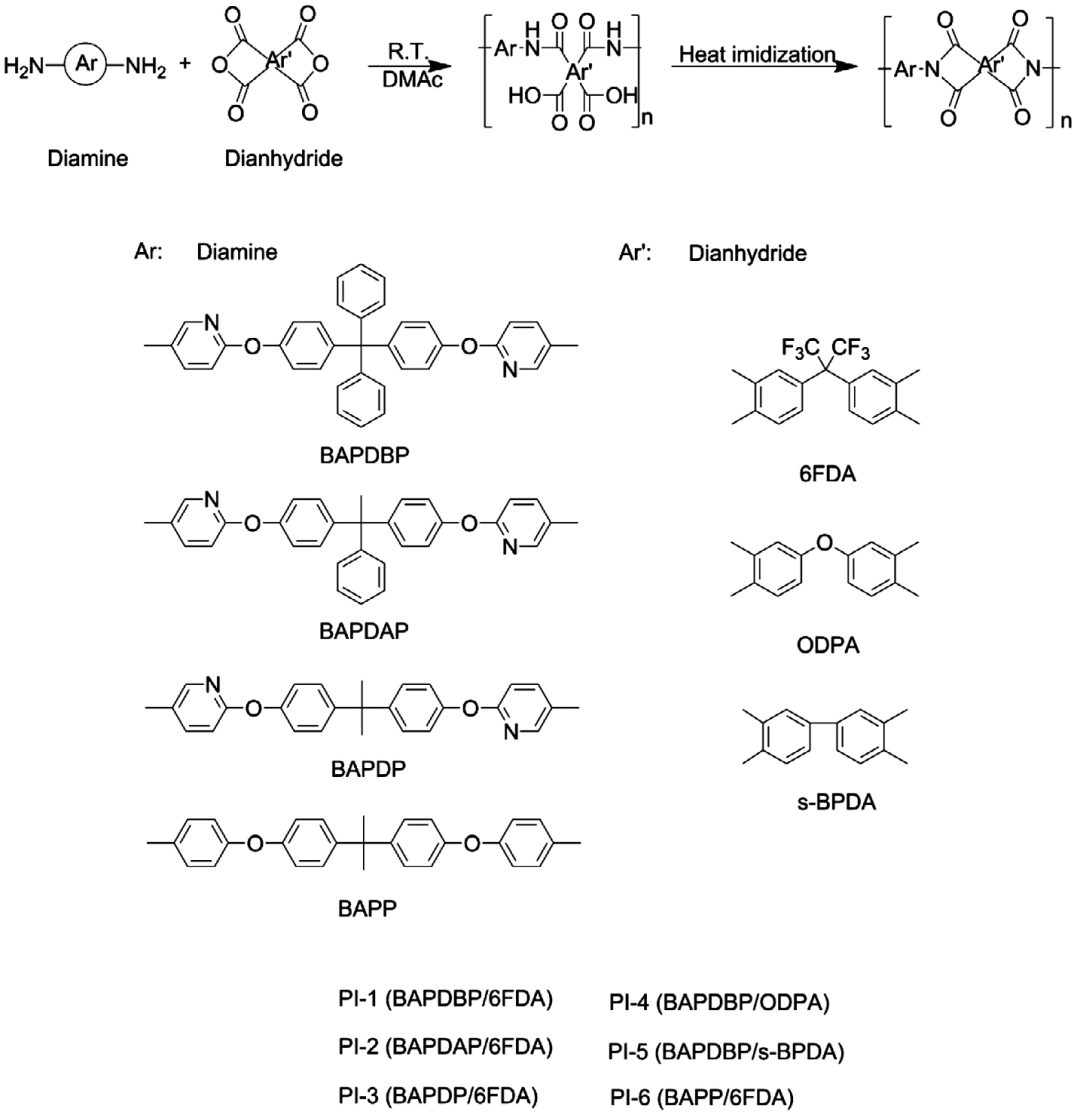
Synthesis route of the polyimides.

PI films were prepared via a traditional two-step method, as shown in Scheme 2. For example, a typical polymerization procedure for the synthesis of poly(amic acid) (PAA) precursors based on 6FDA (PI-1) is as follows. 0.6619 g 6FDA (1.49 mmol) was gradually added to a solution of 0.8000 g BAPDBP (1.49 mmol) in 6 g DMAc. Additional 2.28 g DMAc was then added to adjust the solid concentration of the reaction system to 15 wt% by weight. The mixture was stirred for 12 h at room temperature to give a homogeneous PAA solution. PI films were prepared by casting PAA onto glass plates and then heated in an air oven with a programmed temperature procedure (60 °C/2 h, 80 °C/2 h, 100 °C/1 h, 120 °C/1 h) to remove the solvent. This was followed by an imidization step under vacuum (200 °C/1 h, 250 °C/0.5 h, 300 °C/0.5 h) to produce fully imidized materials. The films were stripped from the plate by soaking in distilled water after they were cooled to room temperature. PI-(2–5) were prepared using a process similar to that described above.

### 2.4. Measurements

#### 2.4.1. Characterization of structures

Hydrogen nuclear magnetic resonance (^1^H NMR) spectra were determined using a BRUKER-300 spectrometer (Massachusetts, U.S.A.) at 300 MHz in CDCl_3_ or DMSO-d_6_.

FT-IR measurements were performed using a Bruker Vector 22 spectrometer (Massachusetts, U.S.A.) at a resolution of 2 cm^−1^ in the range of 400–4000 cm^−1^, with the samples in the form of powders (monomers) and thin films (PIs).

High resolution liquid chromatography-mass spectroscopy (HRLC-MS) data were obtained using an Agilent 1290-microTOF-QII (Bruker, Massachusetts, U.S.A.) high resolution mass spectrometer.

Elemental analysis was run on a Vario EL cube CHN recorder analysis instrument (Langenselbold, Germany).

#### 2.4.2. Inherent viscosities

Inherent viscosities (*η*_inh_) were measured using an Ubbelohde viscometer (Shanghai, China) with a 0.5 g/dL DMAc solution at 25 °C.

#### 2.4.3. Analysis of optical properties

Ultraviolet-visible (UV-vis) spectra of the films were recorded on a Shimadzu UV-vis 2501 spectrometer (Kyoto, Japan) in transmittance mode at room temperature.

#### 2.4.4. Solubility

Solubility was measured by 10 mg of polyimides in 1 mL of solvent at room temperature for 24 h.

#### 2.4.5. Morphology study

Wide-angle X-ray diffraction (WAXD) analysis was conducted using a Rigaku Wide-angle X-ray diffractometer (Tokyo, Japan) (D/max rA, using Cu Kα radiation at wavelength *λ* = 1.541 Å) to determine the morphology structures. Data were collected at 0.02° intervals over the range of 5–50°, and the scan speed was 0.5° (2θ)/min.

#### 2.4.6. Analysis of thermal properties

Dynamic mechanical analysis (DMA) was measured with a TA instrument, DMA Q800 (Delaware, U.S.A.), at a heating rate of 5 °C/min from 50 to 400 °C and a load frequency of 1 Hz in film tension geometry. *T*_*g*_ was regarded as the onset temperature of the storage modulus (E′).

Differential scanning calorimetric (DSC) analyses were performed using a TA instrument, DSC Q100 (Delaware, U.S.A.), at a scanning rate of 10 °C/min under a nitrogen flow of 50 mL/min. To investigate the glass transition temperature, the polyimides samples were heated to a temperature higher than the glass transition temperature to eliminate thermal and stress history.

Thermo gravimetric analysis (TGA) was measured by a TA 2050 (Delaware, U.S.A.), with a heating rate of 10 °C/min under nitrogen and air atmosphere, respectively.

#### 2.4.7. Mechanical measurements

Mechanical properties of the films were measured by a Shimadzu AG-I universal testing apparatus (Tokyo, Japan) with a load of 1 kN at a speed of 5 mm/min. Measurements were performed at 25 °C with film specimens of approximately 30–40 μm thick, 3–5 mm wide and 60 mm long, and an average of at least five individual determinations was used.

#### 2.4.8. Water uptake

Water uptake (WU) of the films was determined by the weight differences before and after immersion in deionized water at room temperature for 24 h, and calculated by the following equation: WU = (*W*_wet_−*W*_dry_)/*W*_dry_ × 100%; where *W*_wet_ is the weight of the film samples after immersion in deionized water, and *W*_dry_ is the initial weight of the samples.

## 3. Results and discussion

### 3.1. Synthesis of monomers

**Figure 1.**
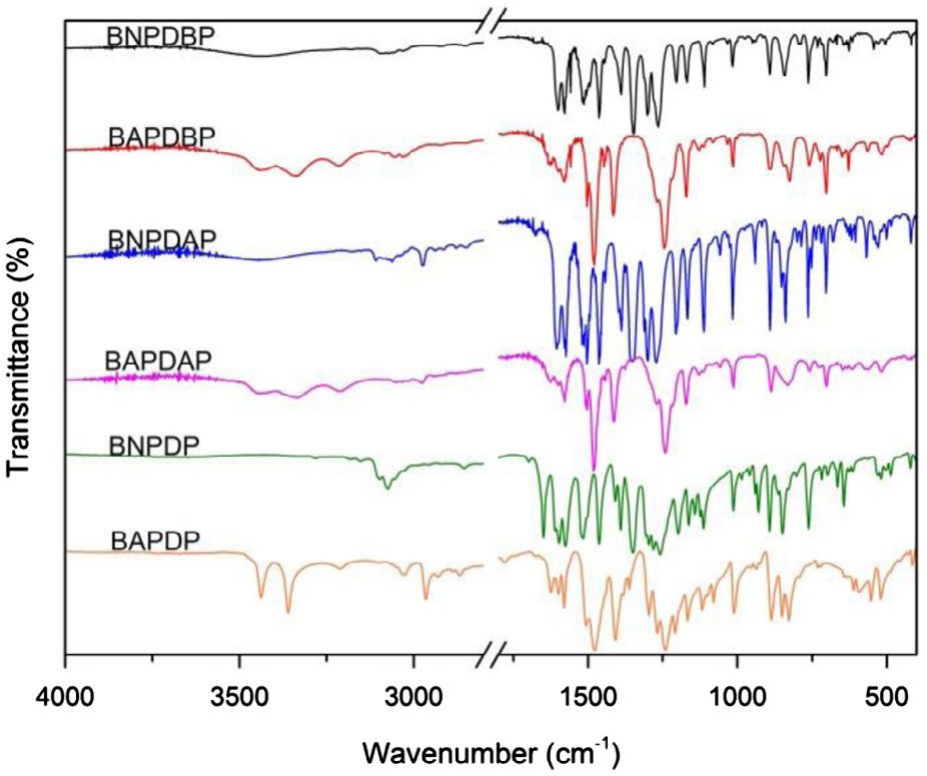
FT-IR spectra of dinitro compounds and diamine monomers.

**Figure 2.**
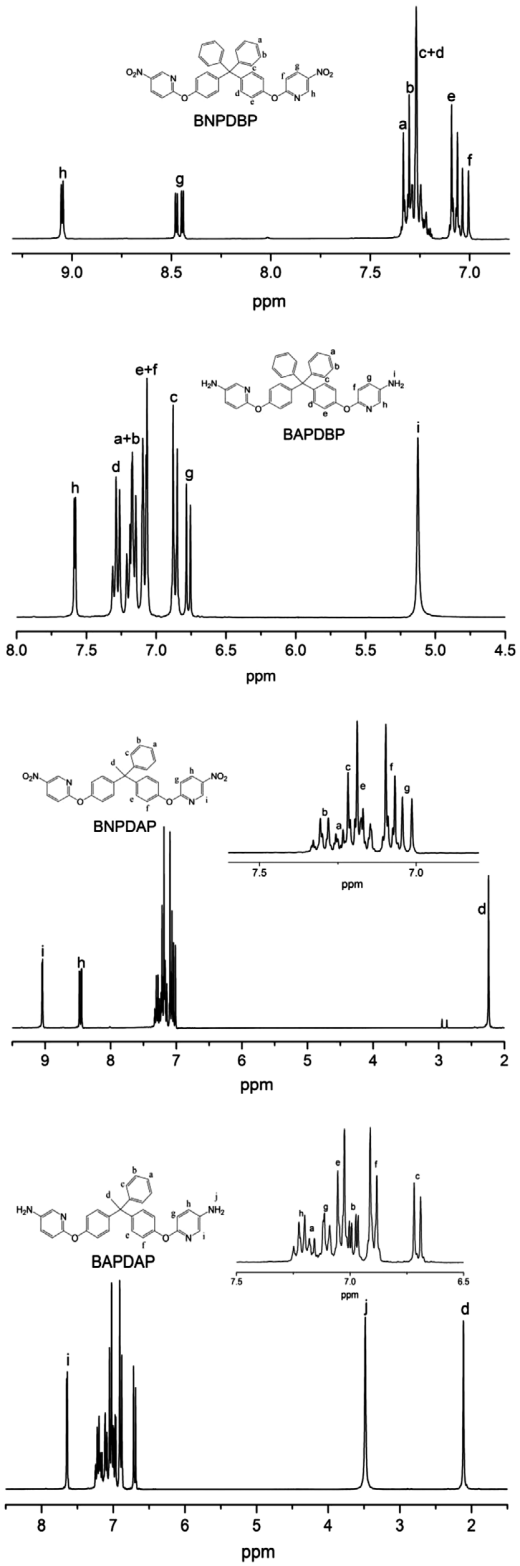
^1^H NMR spectra of dinitro compounds and diamine monomers.

As shown in Scheme 1, BAPDBP, BAPDAP, BAPDP were synthesized by two-step procedures. Firstly, dihydroxy compounds were reacted with 2-chloro-5-nitropyridine using a nucleophilic substitution reaction in the presence of K_2_CO_3_ in the DMF to produce dinitro compounds. Secondly, the dinitro compounds were reduced by Pd/C and NH_2_NH_2_∙H_2_O in dioxane. The analysis results of FT-IR (Figure 1), ^1^H NMR (Figure 2), HRLC-MS demonstrated that all the intermediate and monomers were synthesized successfully.

**Figure 3.**
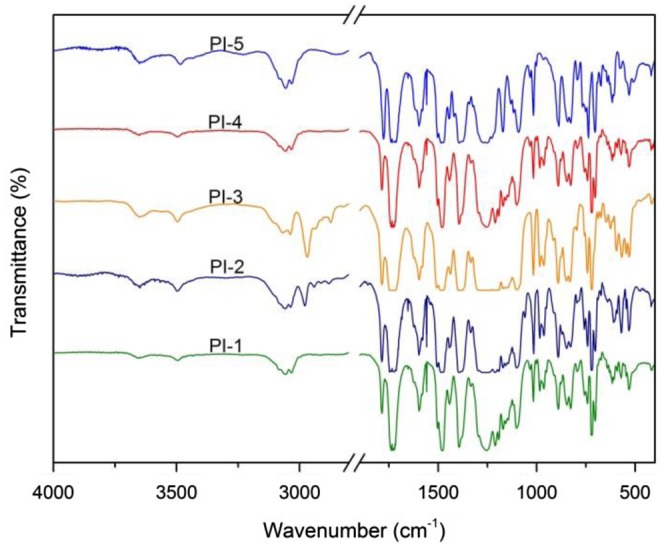
FT-IR spectra of polyimides.

**Figure 4.**
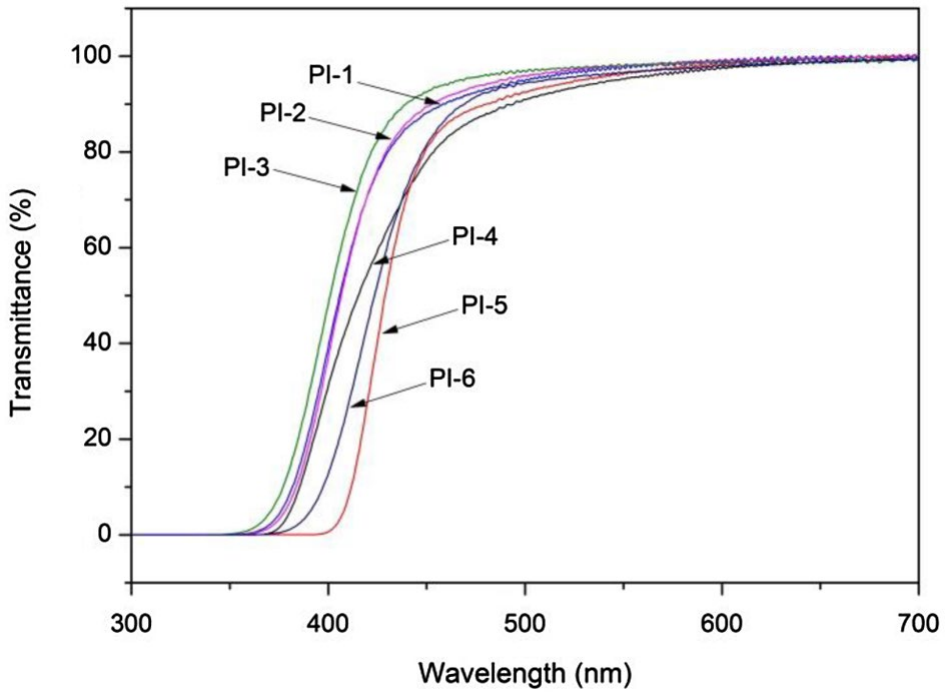
UV-visible spectra of polyimides.

**Figure 5.**
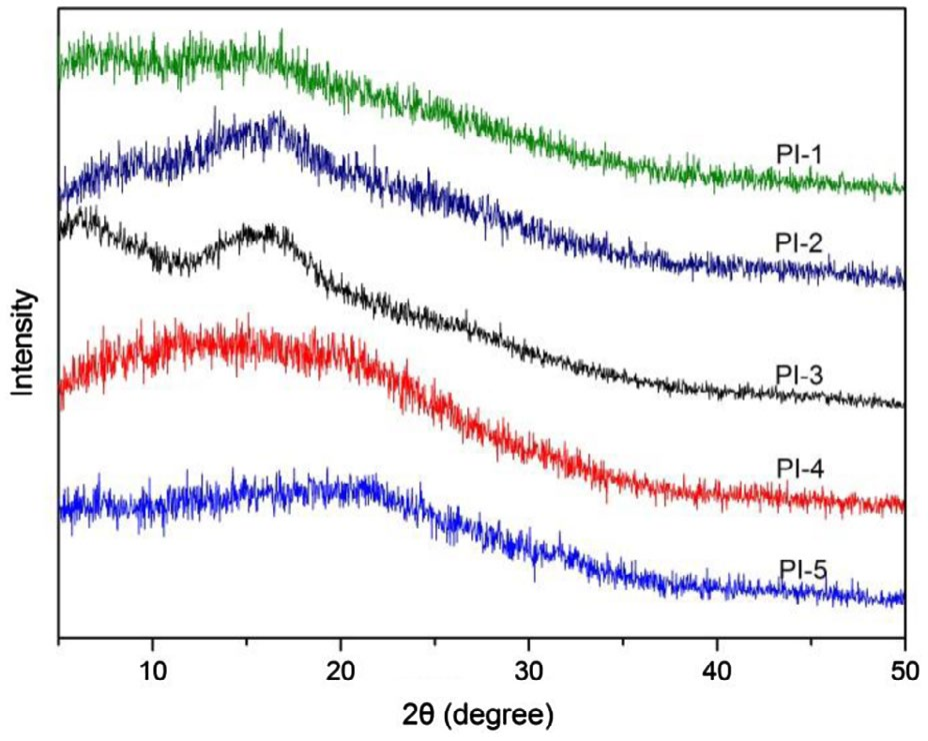
XRD curves of polyimides.

**Figure 6.**
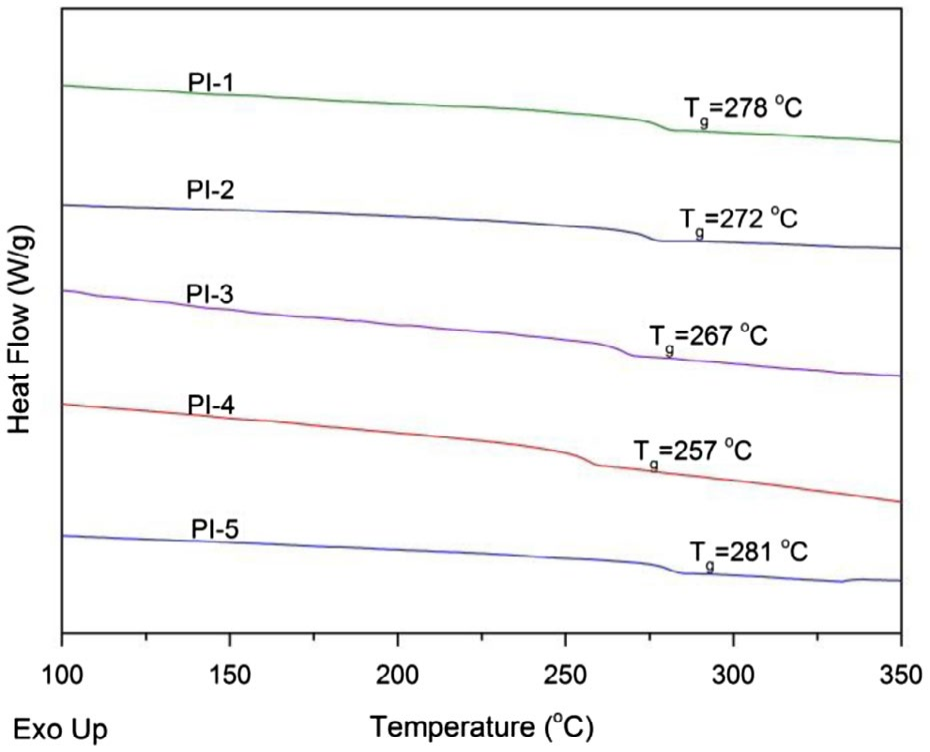
DSC curves of polyimides.

**Figure 7.**
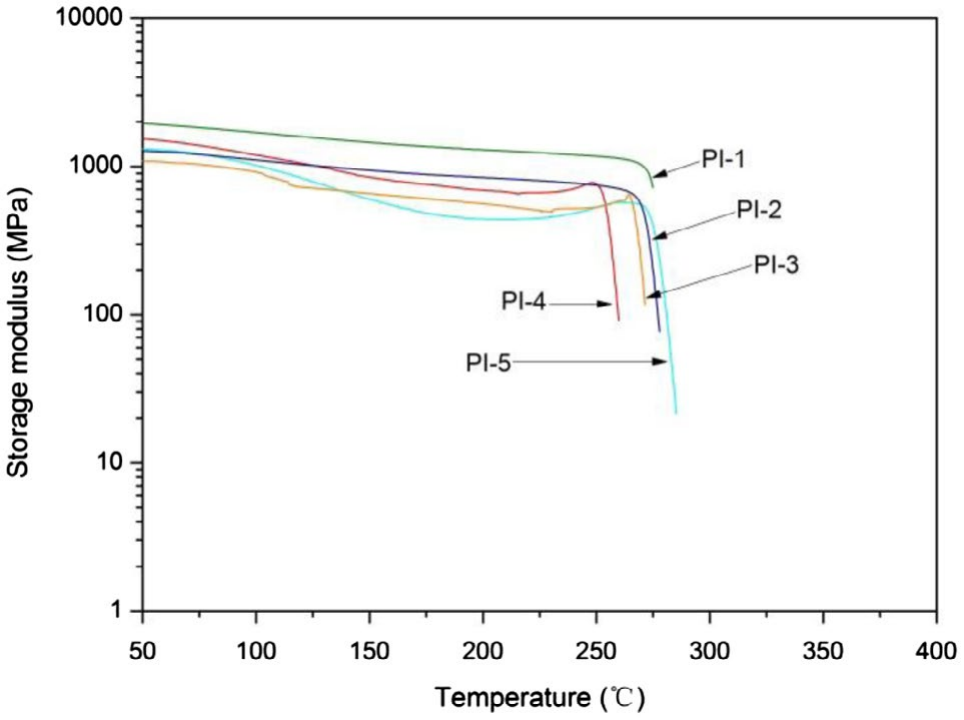
DMA curves of polyimides.

**Figure 8.**
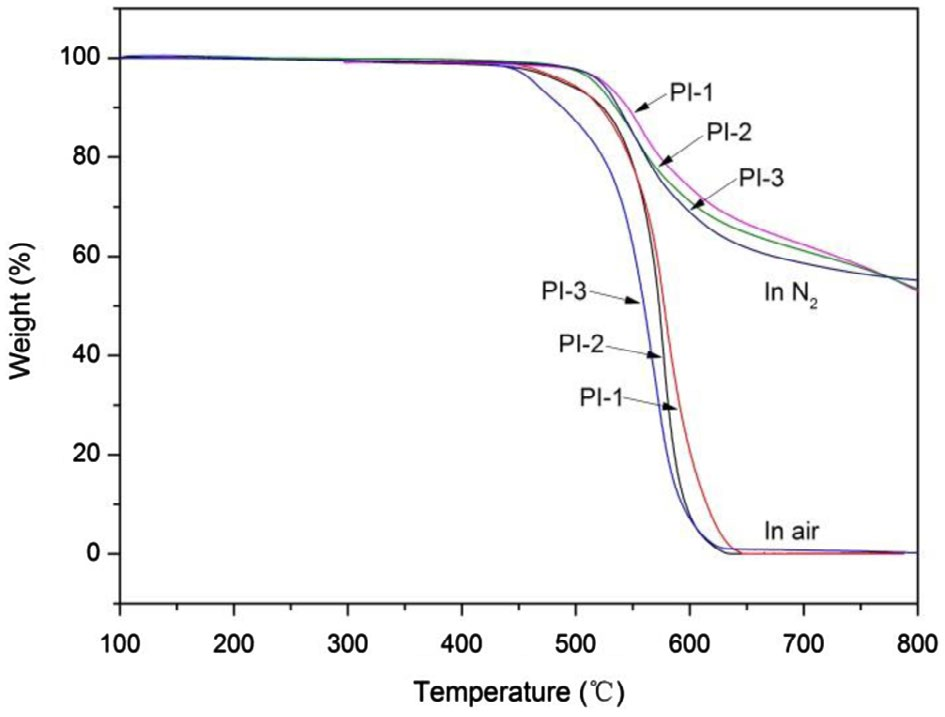
TGA curves of the polyimides (PI-1, PI-2, PI-3) in nitrogen and air.

*BNPDBP* Melting point: 223 °C (DSC peak). FT-IR(KBr): 1601, 1578, 1512, 1494, 1348, 1265, 1205, 1111 cm^−1^; ^1^H NMR (CDCl_3_, ppm): 9.05 (d, H_h_, 2H), 8.48 (dd, H_g_, 2H), 7.33 (m, H_a_, 2H), 7.30 (m, H_b_, 4H), 7.27 (m, H_c+d_, 8H), 7.08 (m, H_e_, 4H), 7.02 (d, H_f_, 2H); HRLC-MS (ESI): 597.5 (M + H)^+^, Calcd 596.6 for C_35_H_24_N_4_O_6_.

*BAPDBP* Melting point: 209 °C (DSC peak). FT-IR(KBr): 3439, 3340, 1599, 1578, 1504, 1479, 1244, 1171, 1014 cm^−1^; ^1^H NMR (DMSO, ppm): 7.58 (d, H_h_, 2H), 7.29 (m, H_d_, 4H), 7.18 (d, H_a+b_, 6H), 7.08 (d, H_e+f_, 6H), 6.86 (d, H_c_, 4H), 6.77 (d, H_g_, 2H), 5.13 (s, H_i_, 4H); HRLC-MS (ESI): 537.5 (M + H)^+^, Calcd 536.6 for C_35_H_28_N_4_O_2_.

*BNPDAP* Melting point: 141 °C (DSC peak). FT-IR(KBr): 2972, 1601, 1578, 1517, 1504, 1352, 1273, 1205, 1113 cm^−1^; ^1^H NMR (CDCl_3_, ppm): 9.04 (d, H_i_, 2H), 8.46 (dd, H_h_,2H), 7.29 (m, H_b_, 2H), 7.24 (m, H_a_, 1H), 7.21 (m, H_c_, 2H), 7.18 (m, H_e_, 4 h), 7.08 (m, H_f_, 4H),7.03(d, H_g_, 2H), 2.24 (s, H_d_, 3H); HRLC-MS (ESI): 535.4 (M + H)^+^, Calcd 534.5 for C_30_H_22_N_4_O_6_.

*BAPDAP* Melting point: 139 °C (DSC peak). FT-IR(KBr): 3438, 3337, 2970, 1598, 1577, 1504, 1269, 1130, 1014 cm^−1^; ^1^H NMR (CDCl_3_, ppm): 7.65 (d, H_i_, 2H), 7.24 (dd, H_h_, 2H), 7.19 (d, H_a_, 1H), 7.10 (d, H_g_, 2H), 7.04 (d, H_e_, 4H), 6.98 (dd, H_b_, 2H), 6.90 (d, H_f_, 4H), 6.71 (d, H_c_, 2H), 3.48 (s, H_j_, 4H), 2.11 (s, H_d_, 3H); HRLC-MS (ESI): 475.5 (M + H)^+^, Calcd 474.6 for C_30_H_26_N_4_O_2_.

*BNPDP* Melting point: 157 °C (DSC peak). FT-IR(KBr): 3070, 2852, 1596, 1576, 1516, 1464, 1349, 1259, 1198, 1115 cm^−1^; HRLC-MS (ESI): 473.4 (M + H)^+^, Calcd 472.5 for C_25_H_20_N_4_O_6_.

*BAPDP* Melting point: 181 °C (DSC peak). FT-IR(KBr): 3438, 3361, 3097, 2966, 1599, 1579, 1477, 1209, 1119 cm^−1^; HRLC-MS (ESI): 413.4 (M + H)^+^, Calcd 412.5 for C_25_H_24_N_4_O_2_.

### 3.2. Synthesis of polyimides

Polyimides based on various diamine monomers (i.e., BAPDBP, BAPDAP, BAPDP) and three commercially available aromatic dianhydrides (i.e., 6FDA, ODPA and s-BPDA) were synthesized using a conventional two-step method, as shown in Scheme 2. The inherent viscosities of the PAA samples measured at 0.38–1.79 dL/g in DMAc at 25 °C are summarized in Table 1. The values of the inherent viscosities (*η*_inh_) tended to be lower than those of highly polymerized PAAs; however, tough and flexible polyimides have been prepared.

**Table 1.** Inherent viscosities of PAAs and elemental analysis of the polyimides.

Code	*η*_inh_^a^ (dL/g)	Elemental analysis (%)				
		Formula of repeating unit		C	H	N
PI-1	0.38	C_54_H_30_F_6_N_4_O6	Calcd	68.64	3.18	5.93
			Found	68.68	3.43	5.38
PI-2	0.73	C_49_H_28_F_6_N_4_O_6_	Calcd	66.67	3.17	6.35
			Found	65.86	3.38	6.54
PI-3	1.79	C_44_H_26_F_6_N_4_O_6_	Calcd	64.39	3.17	6.83
			Found	63.67	3.15	6.88
PI-4	0.41	C_51_H_30_N_4_O_7_	Calcd	75.56	3.70	6.91
			Found	75.88	3.90	6.33
PI-5	0.49	C_51_H_30_N_4_O_6_	Calcd	77.08	3.78	7.05
			Found	77.11	4.02	6.33

^a^ Measured at PAA concentration of 0.5 g/dL in DMAc at 25 °C.

The polymer structure is proved with FT-IR (Figure 3) and elemental analysis (Table 1). FT-IR spectra showed that PAA characteristic absorption bands around 3320–3460 cm^−1^ and 1560–1680 cm^−1^ had disappeared after thermal imidization. The absorptions of the imide ring appeared at 1775–1780 cm^−1^ (asymmetrical C=O stretching), 1720–1730 cm^−1^ (symmetrical C=O stretching), and 1385–1390 cm^−1^ (C–N stretching), which indicated the success of imidization. The elemental analysis data confirmed with the calculated values based on the polymer repeating units.

### 3.3. Optical transparency

Polyimide films based on pyridine and kinky structure showed high optical transparency as presented in Figure 4. The transmittance of polyimides was in the range of 91–97% at 500 nm. It was comparable to the commercial CP films at a nearly thickness which was developed at NASA Langley Research Center [30,31] (Table 2). All the polyimides exhibited lower cut-off wavelength (*λ*_cut-off_) in the range of 353–398 nm. The highly optical transparent are directly related to the kinky substituents which can improve the free volume and inhibited the formation of CTC.

**Table 2.** Optical properties of polyimides.

Code	Film thickness (μm)	Transmittance^a^ (%)	*λ*_cut-off_^b^ (nm)
PI-1	36	95	359
PI-2	36	96	359
PI-3	36	97	353
PI-4	35	91	370
PI-5	37	92	398
PI-6	36	94	374

^a^ Transmittance at 500 nm.

^b^ Cut-off wavelength.

PI films with the same diamine, their optical properties depend on the chemical structures of the dianhydrides. As shown in Table 2, PI-1 showed a relatively higher optical transmittance than PI-4 and PI-5 due to the contribution of –CF_3_ groups in the dianhydrides, which can reduce CT interactions [32–34]. P1-1, PI-2, PI-3 derived from the same dianhydrides showed similarly values of *λ*_cut-off_ and transmittance at 500 nm which should attribute to the long repeat units decreased the influence of phenyl or methyl groups.

PI-3(BAPDP/6FDA) and PI-6(BAPP/6FDA) were synthesized to investigate the effects of pyridine in the chain. PI-3 showed slightly higher transmittance and lower wavelength than PI-6 as listed in Table 2. These results should attribute to the presence of pyridine groups which possess relatively high mole refraction as compared to phenyl unit hence to impact on the *λ*_cut-off_ and transmittance [20].

### 3.4. Solubility

All the fluorinated polyimides showed good solubility in high boiling point polar aprotic solvents, such as NMP, DMAc, DMF etc. and even in low boiling point solvents, such as THF, CHCl_3_ etc.(Table 3) This could attribute to the presence of bulky –CF_3_ groups, which increased disorder in the chains and inhibited dense chain packing, therefore, reducing the interchain interactions to enhance solubility [35]. The kink linkages derived from phenyl pendant can reduce the effects of CTC to some extent. Moreover, the polarizability of nitrogen atom in pyridine in the backbone can improve the solubility [36]. However, PI-4, PI-5 showed a relatively poor solubility than the fluorinated polyimides. PI-4 derived from ODPA containing ether groups are, of course, flexible structure, but has little effect on the chain flexibility or configuration and, in turn, the solubility. As to PI-5, the poor solubility should ascribe to the rigid structure of s-BPDA. The solubility profiles of the polyimides correlated well with the optical transparency data.

**Table 3.** Solubility of polyimides^a^.

Solvent	PI-1	PI-2	PI-3	PI-4	PI-5
m-cresol	+	+	+	−	−
CH_3_COOH	−	−	+	−	−
Pyridine	+	+	+	−	−
DMSO	−	±	±	−	−
DMAc	+	+	+	±	−
NMP	+	+	+	−	±
DMF	+	+	+	±	−
THF	+	+	+	−	−
CHCl_3_	+	+	+	−	−
Cyclohexanone	−	−	±	−	−

^a^ +, soluble at room temperature; ±,swelled slightly soluble in solvent;−, insoluble.

### 3.5. Morphology study

The wide-angle X-ray diffractograms of polyimides are shown in Figure 5. There is no crystallization feature as observed from the wider diffraction peaks, indicating that all of the polyimides showed an amorphous pattern. This correlates well with the thermal analysis. The amorphous behavior of the polyimides is due to the kinky diphenylmethylene linkage, which significantly increased the disorder in the chains and decreased chain packing. In addition, the pendent phenyl groups also decreased the intermolecular forces between the polymer chains, subsequently causing a decrease in crystallinity [37]. Meanwhile, the existence of the pyridyl ether linkage units twist the polymer backbone structure, leading to the formation of amorphous polymer.[38]

### 3.6. Thermal properties

The thermal behavior of the PI films is shown in Table 4. DSC and DMA results revealed the *T*_*g*_ of the polyimides in the range of 257–281 °C by DSC and 254–275 °C by DMA, as shown in Figures 6 and 7, respectively. There is no melting peak in the DSC curves indicates that the amorphous nature of these polyimides which is in accordance with the XRD study. *T*_*g*_ values for BAPDBP based polyimides depending on the structure of the dianhydride component [9] and the stiffness of the polymer chain. The highest *T*_*g*_ was observed for the PI-5 obtained from s-BPDA because of the presence of a rigid chain in the backbone. The lowest *T*_*g*_ of PI-4 can be correlated with the flexibility of ether groups.

**Table 4.** Thermal properties of polyimides.

Code	*T*_*g*_ (°C)		*T*_5%_ (°C)^a^		*T*_10%_ (°C)^a^		*R*_*W*_^b^ (%)
	DSC	DMA	N_2_	Air	N_2_	Air	
PI-1	278	273	525	490	545	523	56
PI-2	272	271	516	494	535	521	53
PI-3	267	266	521	464	537	490	56
PI-4	257	254	519	495	531	527	55
PI-5	281	275	530	489	547	526	49

^a^ Decomposition temperature at which 5% weight loss was recorded by TGA at a heating rate of 10 °C/min under nitrogen and air atmosphere, respectively.

^b^ Residual weight retention at 800 °C under nitrogen atmosphere.

For the structure-property relationship comparison studies, PI-1, PI-2, and PI-3 were prepared from reacting 6FDA with BAPDBP, BAPDAP and BAPDP, respectively. The resulting polyimide film samples gave *T*_*g*_ values in the order of PI-1 > PI-2 > PI-3. This is, because the phenyl substituents are stiffer than the methyl substituents on the backbone. Thus the polyimides with a diphenylmethylene linkage have a higher *T*_*g*_ than polyimides with a phenylethane or methyl linkage.

The thermal stabilities of the PIs were evaluated by TGA under nitrogen and air atmosphere (Table 4). All PI films showed good thermal stability with 5% weight loss temperature above 510 °C and 10% weight loss temperature above 530 °C in N_2_. TGA curves for the 6FDA based polyimides PI-1, PI-2, and PI-3 are shown in Figure 8. While insignificant difference in *T*_5%_ and *T*_10%_ was observed from the TGA curves in N_2_, significant differences were observed from those acquired under air atmosphere. It can be argued that the methyl structure had poor thermal oxidative stability than phenyl pendant.

### 3.7. Mechanical properties and water uptake

The tensile properties of the PI films are summarized in Table 5. All of the polyimides displayed good mechanical properties with tensile strengths of 80–105 MPa, tensile modulus of 1.4–2.6 GPa and elongations at break of 4.3–9.6%. In contrast, PI-1, PI-2, and PI-3, with the introduction of methyl substituents showed lower tensile modulus, which coincided with the thermal analysis results.

**Table 5.** Mechanical properties of polyimides.

Code	*T*_*S*_^a^ (MPa)	*T*_*M*_^b^ (GPa)	*E*_*B*_^c^ (%)	WU^d^ (%)
PI-1	80 ± 0.9^e^	2.2 ± 0.2	4.3 ± 0.3	0.56
PI-2	105 ± 2.8	2.3 ± 0.3	8.8 ± 1.4	0.67
PI-3	88 ± 0.7	1.4 ± 0.2	9.6 ± 2.1	0.60
PI-4	88 ± 1.8	2.6 ± 0.1	5.4 ± 0.2	0.76
PI-5	83 ± 0.7	2.3 ± 0.1	5.0 ± 0.4	0.70

^a^ Tensile strength.

^b^ Tensile modulus.

^c^ Elongation at break.

^d^ Water uptake.

^e^ 0.9 Standard deviation.

Water uptake of the PI films was in the range of 0.56–0.76% at room temperature for 24 h (Table 5). All the polyimides showed very low water uptake compared to the water absorption rate of DuPont Kapton (2.50%) under the same conditions [39]. The results implied that the introduction of pyridine and phenyl pendant in the polymer backbone did not deteriorate the water absorption of the polyimides.

## 4. Conclusions

Two novel diamines containing kinky structure and pyridine were synthesized, characterized, and used for the preparation of a series of polyimides, via a traditional two-step method. All the PI-films showed high thermal stability with the *T*_d5%_ at 516–530 °C and *T*_d10%_ at 531–547 °C in nitrogen and high optical transparency which can be compared to the commercial colorless polyimides. The synergistic effects of kinky structure and pyridine of polyimides leading to high optical transparency without the sacrificing of thermal stability. This is, because the introduction of kinky and bulky substituents which increases the free volume and the pyridine which possess relatively high mole refraction as compared to phenyl unit gave a benefit to improve the optical properties. These properties of polyimides are desirable for application on space solar cells and thermal control coating systems.

## Disclosure statement

No potential conflict of interest was reported by the authors.
